# Adaptive Response of a Gene Network to Environmental Changes by Fitness-Induced Attractor Selection

**DOI:** 10.1371/journal.pone.0000049

**Published:** 2006-12-20

**Authors:** Akiko Kashiwagi, Itaru Urabe, Kunihiko Kaneko, Tetsuya Yomo

**Affiliations:** 1 Department of Bioinformatics Engineering, Graduate School of Information Science and Technology, Osaka University Suita, Osaka, Japan; 2 Department of Biotechnology, Graduate School of Engineering, Osaka University Suita, Osaka, Japan; 3 Graduate School of Frontier Biosciences, Osaka University Suita, Osaka, Japan; 4 Complex Systems Biology Project, Exploratory Research for Advanced Technology, Japan Science and Technology Agency, Osaka University Suita, Osaka, Japan; 5 Department of Pure and Applied Sciences, The University of Tokyo Tokyo, Japan; 6 Complex Systems Biology Project, Exploratory Research for Advanced Technology, Japan Science and Technology Agency, The University of Tokyo Tokyo, Japan; Children's Hospital Boston, United States of America

## Abstract

Cells switch between various stable genetic programs (attractors) to accommodate environmental conditions. Signal transduction machineries efficiently convey environmental changes to the gene regulation apparatus in order to express the appropriate genetic program. However, since the number of environmental conditions is much larger than that of available genetic programs so that the cell may utilize the same genetic program for a large set of conditions, it may not have evolved a signaling pathway for every environmental condition, notably those that are rarely encountered. Here we show that in the absence of signal transduction, switching to the appropriate attractor state expressing the genes that afford adaptation to the external condition can occur. In a synthetic bistable gene switch in *Escherichia coli* in which mutually inhibitory operons govern the expression of two genes required in two alternative nutritional environments, cells reliably selected the “adaptive attractor” driven by gene expression noise. A mathematical model suggests that the “non-adaptive attractor” is avoided because in unfavorable conditions, cellular activity is lower, which suppresses mRNA metabolism, leading to larger fluctuations in gene expression. This, in turn, renders the non-adaptive state less stable. Although attractor selection is not as efficient as signal transduction via a dedicated cascade, it is simple and robust, and may represent a primordial mechanism for adaptive responses that preceded the evolution of signaling cascades for the frequently encountered environmental changes.

## Introduction

Cells alter their gene expression in response to environmental changes or external signals to switch between coherent genetic programs in order to produce a phenotypic state, among many available, that best copes with the new environment. It is increasingly becoming clear that such genetic programs represent attractor states: discrete stable states of gene expression patterns generated by the dynamics of the regulatory interactions between the genes [1]. Small gene networks with mutual regulation of genes can generate multiple attractor states (multistability) and have recently been studied in synthetic [2], [3] or in natural gene networks [4], [5]. These studies have elucidated dynamic properties, including multistability, switch-like behavior, memory effect, oscillation, and robustness in the presence of molecular noise [2], [3], [6]. The presence of multiple attractors has fundamental biological significance, notably in cell differentiation and sympatric speciation [7]–[11]. Specifically, individual attractor states have been suggested to correspond to particular functional cell states or cell types in metazoan [1], [10], [12].

Given the existence of distinct, stable gene expression programs epitomized by the attractor, the question is how cells switch into the appropriate attractor that is commensurate with the environmental condition. For instance, if the nutritional situation requires expression of gene A, how do cells switch into the attrator state in which A is stably expressed? The existing paradigm is that cells have evolved a signal transduction machinery to sense the environmental change and transmit it to the gene regulatory network. In the simplest case, such as in bacteria, the environmental signal may be a metabolite that directly regulates the transcriptional complex that controls the operon involved in its utilization, such as in the case of the lactose operon [13]. In more complex systems, membrane receptor proteins sense environmental changes and trigger a cascade of intracellular molecular events involving “second messengers” or protein modifcation cascades that lead to concerted changes in the expression of several genes. Such molecular signal transduction machineries may have evolved to allow cells to respond rapidly and specifically to frequently occurring changes in the extracellular environment.

However, since the space of environmental conditions is much larger than that of cellular response programs, there is not a program for each condition, and cells need to choose the optimal program for a given condition. Thus, many environmental conditions map onto the same cellular response. Therefore, it is unlikely that cells have evolved a specific signal transduction pathway for every environment it may encounter as in the case of lactose utilization. In fact, infrequently occurring environmental conditions or unspecific, physical perturbations devoid of molecular specificity can evoke specific cellular programs, such as proliferation, quiescence or apoptosis [14]. Some attractor states may provide the optimal gene expression program for the cell to adapt to and cope with a particular, rare environment, yet no specific signaling pathway may exist that connects this rare external condition with the appropriate genetic program. This raises the question: how do cells in the case of rarely occurring environmental changes switch to the adaptive attractor state of the network that expresses the appropriate genes?

Here we use an artificial network with mutually inhibitory operons in *E. coli*, to show that cells possess an inherent ability to adaptively respond to environmental changes by selecting a gene expression state which allows for higher cell activity, without the help of a signal transduction apparatus. Unlike previous work in which genetic switches were linked to fluorescent proteins that report the state of the network [2], [6], [15], we link the bistable switch also to enzymes with phenotypic properties that interact with the environment. We show that cells can select the adaptive attractor and propose that this selection process is a general consequence of the stochastic nature of gene network dynamics. When the network state reaches an attractor that is adaptive, cells exhibit high cellular activity, increasing the turnover rates of mRNAs. This in turn supresses the influence of gene expression noise. In contrast, for the non-adaptive attractor, which accordingly has low cellular activity, the metabolic rate is smaller, and hence, noise overwhelms the deterministic component of the dynamics of the newtork. This causes the cell to be kicked out of the non-adaptive attractor. Thus, the synergism between (i) the deterministic bistable gene expression dynamics, (ii) cellular activity that depends on the match beween gene expression and environmental condition and (iii) stochastic fluctuations in the level of low-abundance molecules, allows the adaptive attractor to be selected.

## Results

### Structure and Properties of the Network with Mutually Inhibitory Operons

**Figure 1:**
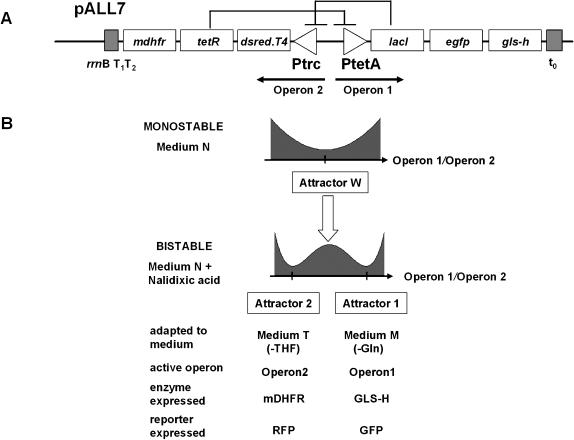
The Plasmid Structures of the Mutual Inhibition Network (A) The structure of the mutually inhibitory operons in pALL7. t_0_ and *rrn*BT_1_T_2_ terminators terminated transcription from Operon 1 and Operon 2, respectively. (B) Summary of the phenotype characteristics of pALL7.

To study adaptive responses to environmental changes without signal transduction machinery, we constructed a synthetic gene network that can exhibit bistability and monitored its ability to switch between two stable attractor states in response to evnironmental change. Gene regulatory circuits that are based on mutually inhibiting operons have previously been reported [2]. However, unlike existing work on synthetic networks (for example, [2], [16]), for our purposes, the network need to express not only state-reporting fluorescence proteins, but also proteins with selectable phenotypes. To obtain a bistable gene network that is linked to metabolic phenotype switching we used the following two mutually inhibitory operons (construct pALL7, Figure 1A). Operon1 is composed of the *tetA* promoter [17], Lac Repressor gene (*lacI*) [13], green fluorescence protein (GFP) gene (*egfp*) [18] and a mutant glutamine synthetase (GLS-H) gene (*gls-h*; the original post-translational regulation was eliminated) [19]. Operon2 is composed of the *trc* promoter [20], Tet Repressor gene (*tetR*) [17], red fluorescence protein (RFP) gene (*dsred.T4*) [21], and the mouse dihydrofolate reductase (mDHFR) gene (*mdhfr*) [22]. The two fluorescent proteins are introduced to monitor the transcriptional level of the two operons. GLS-H and mDHFR confer a metabolic phenotype to each of the two attractor, since these enzymes compensate for two distinct conditions of nutrient depletion.

**Figure 2:**
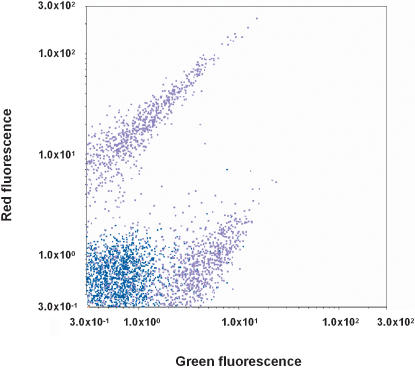
The Three Possible Attractors Generated by the Mutual Inhibition Network under Different Conditions Blue dots represent the expression pattern for cells cultured overnight in Medium N, while purple dots represent that for cells that were transferred from Medium N to Medium N plus nalidixic acid and cultured for 3 days until the number of cells increased sufficiently for flow cytometric analysis. The diagonal correlation for purple dots was not related to any relationship of GFP and RFP expression, but was attributable to the leakage of fluorescence of GFP and RFP to the 600 nm dichroic filter and the band-pass filter at 525 nm±25 nm, respectively.

As a basic property of the circuit architecture, the cells harboring pALL7 can be in a monostable and a bistable behavioral regimes, depending on culture conditions. (Figure 1B and 2). After several serial overnight cultures in Medium N (see Experimental procedures), which does not impose restrictions on essential nutrients, the cells proliferated sufficiently fast so that the gene products of the two operons, including the repressors, were kept low due to dilution. Consequently mutual inhibition was too weak and did not produce bistability. In this monostable regime cells were reproducibly distributed around low levels of expression for both operons (blue dots). Two-color flow cytometry analysis (Figure 2) indicate low levels of expression of Operon 1 (green fluorescence) and Operon 2 (red fluorescence) for each cell. We refer to this state of weak expression from both operons as Attractor W, which is expected to appear at the low total concentration of the two repressors relative to the dissociation constants for their interacton with their promoter binding sites.

In contrast, when the total concentration of gene products is high, the network was shifted into the bistable regime, exhibiting the two Attractors 1 and 2, as expected (Figure 2); either strong expression of Operon 1 with strong repression of Operon 2 (Attractor 1), or vice versa (Attractor 2). This was achieved by slowing down cell proliferation using 170 µg/ml nalidixic acid [23], [24] which reduces the specific growth rate by 30∼40%. In this bistable regime, the balanced state of Attractor W is no longer stable. When either of the operons happens to express its encoded repressor at a slightly higher level than the other, the other operon is slightly suppressed, which in turn decreases the concentration of the repressor for the former operon, leading to a further increase in expression of the former. Thus, the cells are tipped into either of the two self-stabilizing Attractors 1 and 2, as originally intended by the circuit design.

### Shift in Gene Expression Specifically toward the Adaptive Attractors

We next studied the “adaptive” response of the network to external changes by exposing the cells to culture conditions that require the presence of either enzyme (GLS-H or mDHFR) whose mutually exclusive expression is associated with the two attractors. Thus, we ask whether cells can find the “adaptive attractor” that copes with the nutrient condition (Scheme in Figure 1B). For this purpose, we used two environmental conditions to implement the respective nutrient depletion: Medium M lacks glutamine, so that cells are required to syntheszie it to keep up cellular activity. Cells carrying pALL7 can overcome glutamine depletion if glutamine synthetase (GLS-H) in Operon 1 is stably expressed, that is, when they are in Attractor 1. Conversely, Medium T consists of Medium N plus trimethoprim lactate, which causes tetrahydrofolate depletion [22] in the host cells. In this case the host cells carrying pALL7 can overcome tetrahydrofolate depletion if mDHFR in Operon 2 is expressed, which is active when cells are in Attractor 2. In summary, the Attractor 1 (2) is adaptive in mdeium M (T), respectively.

**Figure 3:**
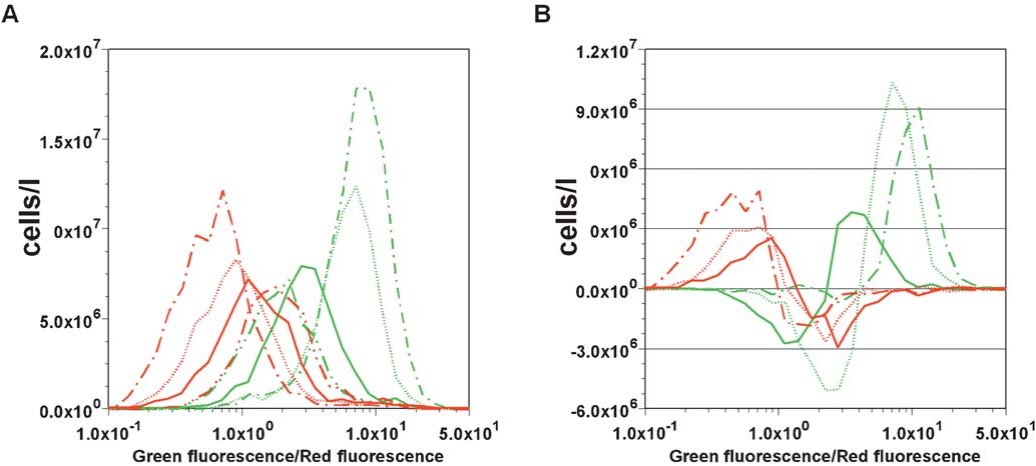
The Initial Time Course of Adaptive Response in Gene Expression (A) Temporal shifts in the distribution of gene expression. After transfer to Medium M cells were sampled at 0.5 h (green dash-double-dotted line), 2 h (green solid line), 5 h (green broken line), and 7.5 h (green dash-dotted line). After transfer to Medium T cells were sampled at 0.5 h (red dash-double-dotted line), 3h (red solid line), 5 h (red broken line), and 7.5 h (red dash-dotted line). Green and red fluorescence of each cell was measured by flow cytometry. By taking the ratio between the two fluorescence intensities, the influence of the diagonal correlation with the slope of 1 shown in Figure 2 could be cancelled. (B) Temporal changes in the number of cells showing the noted fluorescence ratios. The temporal change of the cell number at the denoted ratio on the x axis in Medium M from 0.5 h to 2 h (green solid line) was calculated by subtracting the cell distribution of (A) at 2 h from that at 0.5 h. Similarly, the temporal changes in Medium M were calculated from 2 h to 5 h (green broken line), and from 5 h to 7.5 h (green dash-dotted line). The temporal changes in Medium T were calculated from 0.5 h to 3 h (red solid line), from 3 h to 5 h (red broken line), and from 5 h to 7.5 h (red dash-dotted line).

We put the cells carrying pALL7 to around Attractor W in the monostable regime by overnight culture in Medium N and then transferred them to either Medium M or T supplemented with 170 µg/ml nalidixic acid. In the flow-cytometry measurements, we observed that each cell underwent a unidirectional shift in gene expression pattern toward the attractor expressing the adaptive enzyme in response to respective nutrient depletion (Figure 3A). To monitor the time evolution of the shift we plotted the number of cells per unit volume against the green fluorescence/red fluorescence (G/R) ratio which reflects relative transcriptional activities from the two operons. At 0.5 h after transfer to either Medium M or T, the cells initially retained gene expression patterns, staying around Attractor W. Thereafter, in Medium M the distribution of cells shifted toward Attractor 1. This attractor is adaptive in medium M since glutamine synthetase in Operon 1 is expressed and compensates for the glutamine depletion in this medium. Conversely, the population transferred to Medium T was pulled toward Attractor 2. This shift is also adaptive since the high mDHFR expression compensates for tetrahydrofolate depletion in Medium T. Note that the control transfer to Medium N supplemented with the same concentration of nalidixic acid resulted in a stochastic shift of gene expression toward that of either Attractor 1 or 2 (Figure 2). Therefore, the nutrient depletions in Medium M and T caused a selective, unidirectional shift toward the corresponding adaptive attractors.

### Adaptive Response to Changing Environments

**Figure 4:**
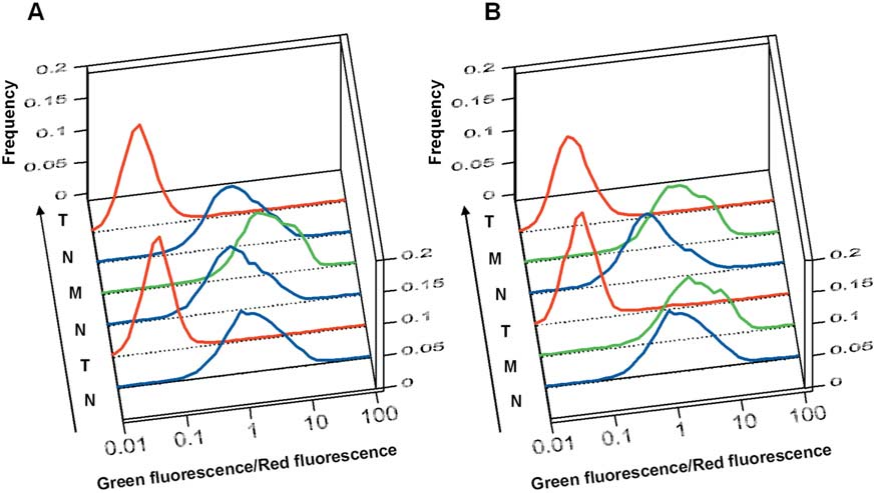
Attractor Selection in Changing Environments *E. coli* OSU1 cells with pALL7 were subjected to serial overnight culture with an inoculum size of 6×10^7^ cells/l every day in changing environments. (A) Days 1–5 in Medium N, days 6–7 in Medium T, days 8–10 in Medium N, days 11–13 in Medium M, days 14–15 in Medium N, and day 16 in Medium T. On the last day of serial overnight cultures in the same medium, the cells were subjected to flow cytometric analysis. (B) Days 1–5 in Medium N, days 6–7 in Medium M, days 8–9 in Medium T, days 10–11 in Medium N, days 12–13 in Medium M, and day 14 in Medium T.

We next examined how cells adjust their gene expression adaptively to fluctuating environments. Cells carrying pALL7 were subjected to serial overnight culture with sequential medium changes in two different orders (Figure 4A and 4B). In these experiments, nalidixic acid was omitted from all three media to minimize the risk of unexpected genomic rearrangements during long-term cultivation. When cultured in Medium N, most of the cells exhibited a neutral position of G/R, *i.e.*, were in Attractor W. Following transfer to Medium T (Figure 4A), the cells displayed a low G/R, indicating strong expression of Operon 2, encoding mDHFR, which compensates for the depletion of tetrahydrofolate in this medium. After return to Medium N, the cells again displayed the neutral position. The cells showed successive changes in G/R: neutral→high→neutral, when the medium was changed in the order N→M→N; consistently, the high G/R ratio here indicated expression from Operon 1, which encodes glutamine synthetase that compensates for the lack of glutamine in Medium M. Further transfer to Medium T caused the G/R ratio to shift back to the low value, indicating that the cells retained the ability to adjust their gene expression adaptively to Medium T. When the medium was changed in a different order (Figure 4B), the same three distributions appeared in response to the three media.

**Figure 5:**
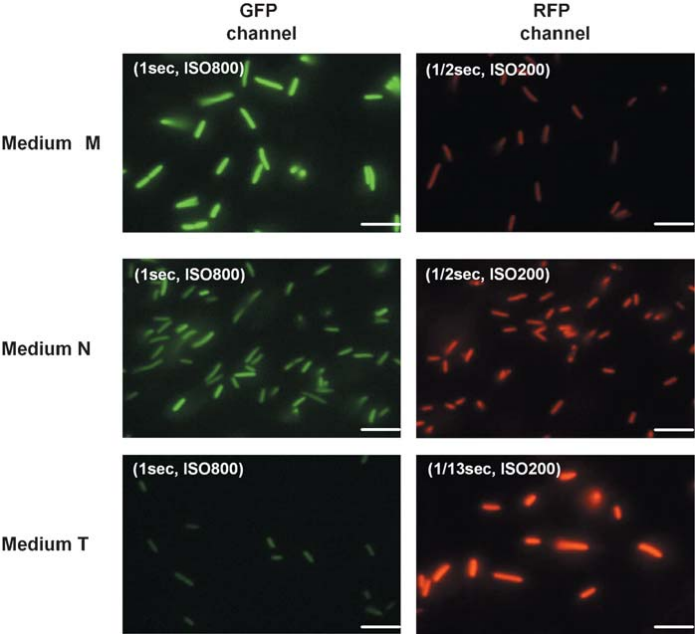
Microscopic Examination of Cells Cultured Overnight in Three Different Media GFP and RFP fluorescence of cells in Medium M, N, and T were examined using an Olympus IX70 microscope and a KEYENCE VB-6010 CCD camera. For the GFP channel, a BA470-490 excitation filter, DM505 dichromatic beam splitter, and BA515-550 emission filter were used. For the RFP channel, a BA520-550 excitation filter, DM565 dichromatic beam splitter, and BA580IF emission filter were used. The exposure time and the sensitivity expressed as ISO values are shown in the pictures. Scale bar, 5 µm.

Microscopic analysis confirmed that at the single cell level these reproducible distributions with the peaks of high, neutral, and low G/R correspond to Attractor 1, Attractor W, and Attractor 2, respectively (Figure 5). The cells in Medium N expressed GFP and RFP weakly, indicating Attractor W. On the other hand, cells in Medium T showed strong expression of RFP and repression of GFP compared to those in Medium N, indicating Attractor 2. Similarly, cells in Medium M exhibited strong expression of GFP and repression of RFP, consistent with Attractor 1. The alternative appearance of the three attractors in each medium was also supported by mRNA quantification; the average numbers of mRNA copies transcribed from Operon 1 and Operon 2 per cell were 10.0 and “undetectable”, respectively, in Medium M, whereas they were “undetectable” and 6.0, respectively, in Medium T, and “undetectable” and 0.3, respectively, in Medium N. These results clearly show that the cells chose the adaptive attractors reproducibly in fluctuating environment.

### The Response Is Not Due to Proliferation of Fit Cells

A fundamental question of cell state switching is whether (i) an external signal that causes a commitment to the expression of a particular phenotype does so by somehow instructing the gene transcription apparatus to express the appropriate (set of) gene(s) in all cells, or (ii) the signal merely promotes survival and expansion of the few cells that “happen” to express that desired phenotype [25]. In other words, if the switching is only due to the scenario (ii), the two attractors are selected with equal probability first but only the cells in the adaptative attractor will proliferate, causing a shift in the population distribution. We found that the scenario (ii) alone cannot explain the observed macroscopic shift toward adaptive attractor, but the scenario (i) indeed is necessary, as demonstrated by careful monitoring of the time course in which cells redistribute between each of attractors, as explianed in the following.

At 0.5–2 h, when the total cell concentration had hardly increased as indicated by the minimal increase in the total areas under the curve (AUC) of distribution (Figure 3A), the cell distribution began to shift toward the adaptive attractor. To better represent the shift in population (change in histogram of cell number for a given G/R ratio) Figure 3B shows the increment in cell number within each bin in the histogram up to each sampling time, obtained by subtratcing the cell number from that at the previous sampling time point in Figure 3A. In Medium M, the subpopulation of cells with high G/R ratio clearly increased within the time period from 0.5 to 2 h (1.5×10^7^ cells/l in the G/R ratio between 10^0.3^ and 10), while that with low G/R ratio decreased (1.2×10^7^ cells/l in the G/R ratio between 10^−0.5^and 10^0.3^). The small difference in the total AUC between the downward peak for cells with low G/R ratio and the upward peak for cells with high G/R ratio indicates little cell growth from 0.5 to 2 h. Since nutrient starvation for less than 5 h caused little cell rupture (data not shown), the two allmost equal AUCs of downward and upward peaks indicate that each cell underwent a unidirectional alteration in its gene expression pattern from Attractor W toward Attractor 1 during this time period. Taken together, this kinetic analysis suggests that the observed unidirectional shift until 2 h can be attributed not to the prolification of cells randomely tipped toward the adaptive attractor (scenario (ii)) but to a deterministic change in gene expresssion of each cell (scenario (i)).

In contrast, for the period from 2 to 5 h, the change in gene expression toward Attractor 1 and the growth of cells at high G/R occurred simultaneously, as indicated by the smaller total area of the downward peak for cells with low G/R ratio compared with that of the upward peak for those with high G/R ratio. From 5 to 7.5 h, as indicated by the small downward peak for cells with the low G/R ratio, the change in gene expression toward Attractor 1 was almost complete, while cells with the high G/R ratio continued to grow. Thus, the time course analysis indicates that after exposure to Medium M the cells that happened to have been tipped toward the adaptive attractor at the beginning were pulled to these attractors and started growing in the favorable condition (scenario (ii)) while the other cells that happened to have been tipped to the non-adaptive attractor escaped from them and switched to the adaptive attractor in a scenario (i) process. Upon exposure to Medium T, the cells showed the same time course of attractor selection but in the opposite direction. These unidirectional changes in gene expression suggest that cells are capable of response adaptively to environmental changes through attractor selection. Thus, within the limits of measuring the time course of gene expression for each cell, both mechanisms appear to play a role. It remains to be determined to what extent each of two scenarios contribute to the observed adaptive attractor selection.

### Fitness-Induced Gene Expression without Signal Transduction Machinery

Given that the mechanism of regulation of selecting the attractor cannot be solely explained by selective growth advantage of randomly swicthed cells, one may suspect that adaptive attractor selection in response to nutrient depletion may be due to some direct but ‘hidden’ instructive signal transduction that may somehow be “hard-wired” in the host cell's genome, connecting nutrient situation to the respective cis-region of the two metabolic genes. To exclude this possibility, we carried out a ‘promoter swap’ experiment. The *mdhfr* and *gls-h* genes were swapped in the pALL7 plasmids, so that the same regulatory circuit was maintained (plasmid pALL8, Figure S1A). This new plasmid was introduced into the same host cells, *E. coli* OSU1. The transformed cells were subjected to a series of overnight cultures in essentially the same media with a slight modification (legend of Figure S1). As shown in Figure S1B, attractor selection took place in the same way with the mutually inhibitory network of the pALL8 plasmid as with the pALL7 plasmid but only with the difference that with pALL8, the correlation between the media and the strongly expressed operon was the reverse of that observed with pALL7.

We did not observe a non-adaptive response with pALL8, in that for instance, depletion of tetrahydrofolate would have activated Operon 2, which contains the promoter that drives mDHFR in pALL7 but glutamin synthetase in pALL8. Thus, the promoter swap experiments corroborate the principle that expression of operons was dependent on the fitness conveyed by the encoded enzyme to the environmental condition and was not due to some unexpected fixed functional relation (such as an unknown signaling pathway) between the DNA sequence of these nominally generic promoter regions and the environmental signal.

In summary, we have shown experimentally that by introducing a gene network with bistable attractors expressing phenotypes that are sensitve to the environment, most of the cells changed the attractor state and associated gene expression in response to the environment, so as to mount an adaptive response by producing the enzymes necessary to compensate for the nutrient depletion.

### Theoretical Model for Adaptive Attractor Selection

How can we understand the above adaptive attractor selection without specific signal transduction machinery? Here, we propose that such selection is a rather general process allowing cells to grow by selecting and maintaining stochastic gene expression patterns. The basis for the alternative expression patterns in the present gene network is a deterministic bistable system. Thus, we propose here first a model for bistability for our network that links attractor state with a metabolic phenotype.

We analyzed a standard model of mutually inhibitory operons which can generate bistability [2], but introduced terms to capture the phenotypic consequence of a stable state, which determines the adaptation to an environment, and a noise term:
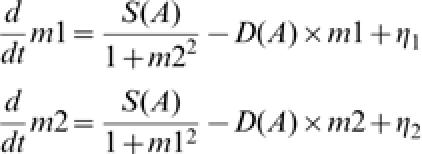
where *m*1 and *m*2 are the concentrations of the mRNAs or their protein products (which are here lumped together into one term since the phenotype switch of the population occurs at a slower time scale than transcription), transcribed from Operon1 and Operon2, respectively. *S*(*A*) and *D*(*A*) are the rate coefficients of synthesis and degradation and/or dilution due to the cell volume growth, respectively. Importantly, they depend on *A*, which represents cellular activity. *η*_1_ and *η*_2_ represent independent white noise in gene expression.

By setting the equations (1) without noise to d*m*/d*t* = 0, one can obtain the fixed-point solutions for the system. This yields a single attractor of *m*1* = *m*2* for *S/D<0.5* (corresponding to Attractor W in the monostable regime in the experiment in the absence of nalidixic acid). For *S*/*D*>0.5, there are two attractors satisfying (*m*1 = m*, *m*2 = 1/m*) and (*m*1 = 1/m*, *m*2 = m*) (see Supporting Information). Without the noise term, the initial condition with *m*1>*m*2 is attracted to the attractor located in the region of *m*1>*m*2 and vice versa.

To capture the phenotypic consequence that allows adaptation we introduce the variable *A* = “cellular activity”, which is increased when cells approach the adaptive attractor, expressing the genes that allows survival and optimal growth in a given environment. It is not easy to define “cellular activity” on a material basis, as it is a complex function of the concentrations of ATP and other chemicals. However, cellular activity can be lumped together and represented as the variable *A* that increases monotonously with cell growth rate, particularly for unicellular organisms.

The first important postulate in the model is that both synthesis *S*(*A*) and degradation and/or dilution *D*(*A*) are increasing functions of activity *A* (albeit in distinct ways) which in turn is correlated with the nutrition condition and growth rate. Since the rate of metabolic processes increase with activity *A*, the amplitudes of the synthesis rate of mRNA or its protein product, *S*(*A*), is expected to increase with *A*. As the degradation rates of LacI and TetR are much smaller than the specific growth rate of cells in this work [26]–[29], the amplitude of *D*(*A*) is mostly determind by the dilution due to cell growth and thus increases with *A*.

The different attractors show different activities. The adaptive attractor has higher activity, i.e., larger values of *S*(*A*) and *D*(*A*). In other words, the deterministic part of Eq. (1) (i.e., all terms without the noise terms) take larger (smaller) values for an adaptive (non-adaptive) attractor, respectively. (Recall *S*(*A*) and *D*(*A*) are increasing functions of the activity *A*).

We now discuss selection of adaptive attractor. First we note that such selection of attractors is not possible in the above deterministic dynamics alone. However, gene expression is always accompanied with considerable random fluctuations, or noise [30], represented by the term *η* which can account for noise-driven transitions between attractor states [31] .

The second basic postulate in the model is that the noise amplitude is independent of the activity *A*, or at least it does not vanish with the decrease of the activity. Specifically, in a first approximation we assume a constant amplitude of noise. However, the specific form of noise in the Langevin Eq. (1) is not important, as long as a considerable amount of noise remains when the network is in the non-adaptive attractors, even if noise strength may depend on (*m*1, *m*2), or activity to some extent.

In general, for noise arising from chemical reactions associated with the activity, its strength should increase with the activity. However, even if the activity vanishes, fluctuation in gene expression may remain due to basal (housekeeping) biochemical processes in the cell. Indeed, recent measurement of noise on a variety of environmental conditions suggests that considerable amount of noise remains independent of the growth speed of a cell that is correlated with cellular activity [32]. Hence, it is natural to assume that some part of noise is activity independent, even though the actual form of noise strength is difficult to predict at the present stage. In our experiment, we found that considerable noise remains even for a cells in the “non-adaptive condition” where they are in a state with low activity. When cells located around Attractor 1 in Medium M were transferred to Medium T (Figure 4B), most of the cells moved to Attractor 2, while a small fraction of cells remained at Attractor 1. The observation that the latter cells, though they had very low activity around the non-adaptive attractor, maintained a similar scattering in fluorescence intensity (Figure S2B,) suggests that noise is present even in states with low activity. On the other hand, we found that the relative contribution of noise to the deterministic metabolic rate decreased as cells approached the adaptive attractors. In Figure 3A, the distribution of the G/R ratio became narrower as cells shifted their gene expression toward the adaptive attractors. The relative variances in G/R ratio (estimated as full width at half-maximum of the histogram) decreased by 72% (in Medium T) or 57% (in Medium M) by the time of 7.5 h, suggesting that the relative magnitude of the noise term decreased while that of the deterministic metabolic term became large with increasing activity.

We now describe how, given the above two basic postulates, the adaptive attractor is selected. Let us first consider the case in which an environmental change causes a marked decrease in *A* because the gene expression pattern is inappropriate (i.e., the cell is in the non-adaptive attractor). Then, the deterministic metabolic rate in Eq. (1) will be so small that it will approach the same magnitude as that of the noise term, *η*. The dynamics of gene expression will therefore be dominated mostly by random fluctuations. This is true as long as the network is in a region with the state of low activity. On the other hand, when the network moves into a region with high acttivity *A*, *i.e.*, to the adaptive attractor, then the metabolic rate inceases, and the deterministic part becomes much larger than the noise term. Consequently, the dynamics of the system will be governed by the deterministic part of Eq. (1). Thus, whichever gene expression state the network starts with, gene expression will continue to fluctuate until it arrives at the adaptive attractor which is more stable against the noise because of the relatively lager metabolic rate of the first and second terms in Eq. (1).

**Figure 6:**
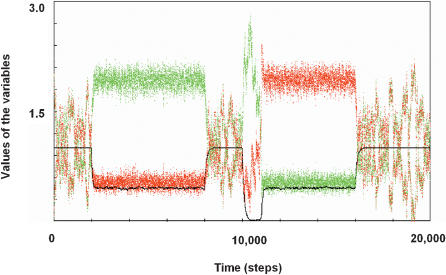
Simulation of Dynamics of Networks with Mutually Inhibitory Operons Black, green, and red indicate activity *A*, and the expression levels at the two operons, *m*1 and *m*2, respectively. Culture conditions were captured by *N*1, *N*2 (see Supporting Information). We employed a changing environment: (*N*1, *N*2) = (10,10) from 0 to 2000 step, (0,10) to 8000, (10,10) to 10000, (10, 0) to 16000, (10,10) to 20000. The parameters used in this simulation were: *S*(*A*) = 6*A*/(2+*A*) and *D*(*A*) = *A*, *N*_*thr*_1_ = *N*_*thr*_2_ = 2, *P* = *C* = 0.01, and *n*1 = *n*2 = 5. Initially, the environment contained both nutrients at sufficient levels from *t* = 0 to 2000, and activity *A* was less than but very close to 1. Then, following the change in the environment causing depletion of *N*1 = 0 but with *N*2 = 10, the system started to lose the activity, which destabilized the Attractor W, and entered the regime with two attractors, where the attractor with higher levels of *m*1 is selected. When returned to the nutrient-rich environment at *t* = 8000, the system came back to the single attractor with *m*1 = *m*2 = 1. Then, the environmental conditions were changed to depletion of the other nutrient, *N*1 = 10, *N*2 = 0 at *t* = 10000, and the system first deviated to express more of *m*1 but less *m*2. This deviation to the side of the non-adaptive attractor occurred by chance, but the small value of *m*2 did not compensate for the nutrient depletion and the activity is reduced close to zero. Then, the noise term in equations (1) took over in influence on the system. This fluctuation decreased only when *m*2 happened to rise sufficiently to allow activity to increase again at around *t* = 11000.

With a simple form of *D*(*A*) and *S*(*A*), the above dynamics for attractor selection was confirmed by numerical simulation of Eq. (1) (Figure 6). (for details on the mathematical model, see Supporting Information).

The present scheme of adaptive attractor selection generally works for a system with dx/d*t* = f(x, *A*)+*η*, with f(x, *A*) = {f_1_(x, *A*),f_2_(x, *A*),…} where f_i_(x, *A*) is the metabolic rate of mRNA or protein for gene i. In general, if |f(x, *A*)| increases with *A*, the attractor with small *A* is disturbed by noise so that an attratcor with high *A* is selected.

Switching between attractors has been studied in dynamical systems as noise-induced selection of attractors [33], [34]. In these previous studies, however, the direction of switching was not adaptive. In contrast, in the case presented here, due to the coupling of the attractor states with the cellular activity which influences the rate of the turnover of the deterministic state variables, an adaptive attractor with its higher cellular activity is inevitably selected in the presence of noise.

## Discussion

### Perspective for Genomic Networks

We propose here the concept of attractor selection based on experimental and theoretical studies on a simple network with mutually inhibitory operons in which the network autonomously chooses the appropriate gene expression pattern associated with the adaptive attractor in response to environmental changes that are not mediated by a cognate signal transduction machinery. This mechanism for environmental adaptation of gene expression crucially depends on the ratio of the metabolic rate (mRNA, protein turnover) controlled by the regulatory circuit and on the presence of noise in gene expression. The latter is relatively high compared to the deterministic regulation when cells are in unfavorable conditions so that the overall metabolic regulation is weak due to low cellular activity. Thus, the ratio of (deterministic) metabolic regulation relative to noise is reduced or increased around non-adaptive or adaptive attractors, respectively. As long as this correlation is satisfied, network structures other than the simple network investigated here will be able to respond to environmental changes through attractor selection if they possess multiple attractors [theoretically confirmed by Furusawa and Kaneko, unpublished]. Thus, noise-driven selection between two alternative attractor states differs from previously studied decision between functional states, such as glucose/lactose utilization in *E. coli* or lysogeny/lysis in lambda phages, in that there is no explicit, specific molecular link between proteins of the gene network and an external signal (metabolite as allosteric regulator of a key regulatory protein; UV light, etc.).

Our simple regulatory circuit utilizes common promoters and their repressors. However, the noise in gene expression from plasmids does not appear to differ markedly from that of the genomic genes [35], [36]. The existence of attractor selection in genome-encoded networks should be investigated in future studies, although the response of the whole genomic network in terms of attractor selection will be not as simple as that described here as it will involve combinations of many molecular mechanisms, including canonical signal transduction pathways.

### Comparison between Signal Transduction and Attractor Selection

Signal transduction is a basic mechanism for cellular responses to environmental changes, which is based on complex networks that connect the environment to genome. Each signal transduction network must have evolved through natural selection by responding to environmental changes that have occurred frequently in the past. Signaling pathways then have been conserved because they allow the host cells to adapt rapidly to specific environmental changes.

However, in addition to such frequently occurring environmental changes, unicellular organisms may encounter a large number of different starvation conditions each of which may appear only a few times during their lifespan. In addition, cells may not have evolved an attractor state to every environmental condition, yet some may confer better adaptation than others. It is then hard to imagine that the cells would have evolved and maintained specific signal transduction machineries for all possible rare environmental changes so as to map every environment to an optimal attractor, because of the high cost of maintaining all these signaling machineries. Even for frequently occurring environmental changes, attractor selection can be beneficial for cells since it requires no specific signal transduction apparatus, if speed of state transition is not an issue.

Due to its stochastic nature, attractor selection may not be as efficient as ordinary signal transduction, but it may prevent cells from dying in fluctuating environments. Molecular signal transduction may have evolved for efficient adaptation, but in view of our present results it is plausible that attractor selection may still be utilized as a primitive adaptation for sustainability in broadly changing environments. Attractor selection may facilitate the design of a network that can robustly respond in an adaptive manner to unknown environmental changes without requiring a large number of specific sensors and transducers. It may also be viewed as a sort of Darwinian preadaptation for the evolution of signal-specific transduction pathways when a particular new environmental condition becomes dominant and hence contributes to evolvability.

## Materials and Methods

### Plasmid construction

All plasmids were constructed using standard molecular cloning techniques [37]. Genes and promoters were obtained from the following sources: Ptrc from pTrc99A (Amersham Biosciences); PtetA from pASK-IBA3 (Sigma Genosys); *lacI* from pTrc99A; *tetR* from pcDNA6/TR (Invitrogen); *egfp* from pEGFP (BD Biosciences Clontech); *dsRed.T4* (a gift from Dr. B. S. Glick, The University of Chicago) [21]; *mdhfr* from pQE16 (Qiagen); and *gls-h* from pKGN-H [19]. The plasmid contained the ColE1 origin of replication and ampicillin resistance gene. The t0 terminator from pPROTet.E333-lacZ (BD Biosciences Clontech) and *rrn*BT_1_T_2_ terminator [38] from pTrc99A were used to terminate transcription from Operon 1 and Operon 2, respectively.

### Strain, media, and growth conditions

*E. coli* strain OSU1, a derivative of DH1 [39], was constructed by replacing the *glnA* gene with the *cat* gene by homologous recombination [40]. For cells carrying pALL7, the following three media were employed. 90 mg/l ampicillin was added to Medium C to prepare Medium M [41]. Medium N consisted of Medium M plus 90 µM glutamine, which compensates for the lack of the *glnA* gene in the genome of OSU1. Medium T was Medium N plus 580 mg/l of trimethoprim lactate, which completely inhibits bacterial DHFR and requires a high level of expression of mDHFR for survival of *E. coli* cells. To match the effective strength of PtetA to that of Ptrc, anhydrotetracycline, which inhibits Tet repressor, was added to the three media at equal concentrations of 0.5 µg/l. All culture steps were conducted at 37°C with aeration after inoculation of 6×10^7^ cells/l, unless otherwise noted. The number of cells was measured using a Particle Analyzer SD-2000 (SYSMEX). The cells from each population were stored at −80°C before flow cytometric analysis.

### Flow cytometric analysis

The samples stored at −80°C were thawed at 25°C in a water bath and kept on ice before analysis. The single-cell fluorescence measurements were carried out on a COULTER^®^ EPICS® ELITE Flow Cytometer (Beckman Coulter) with a 488-nm Argon excitation laser and band-pass filters of 525±25 nm for green fluorescence and a 600 nm dichroic filter for red fluorescence. All flow event data were converted to text format using WinMDI Version 2.8 and analyzed with MATHEMATICA 5.0 (Wolfram Research) and DeltaGraph Version 5.0.1.

### mRNA measurement

Total bacterial RNA was prepared with RNeasy kits (Qiagen). Northern blotting and hybridization were performed according to standard methods and visualized with the AlkPhos Direct™ Labeling and Detection System (Amersham Biosciences). Double-stranded *lacI* and *dsred.T4* DNAs were used as probes for transcripts from Operon 1 and Operon 2, respectively. Hybridization signals for the mRNAs from Operon 1 and Operon 2 were quantified using ImageJ 1.29.

### Model analysis

The numerical simulation of the Langevin equation (1) was conducted by the Runge-Kutta method using a program developed in Microsoft Visual C++, as well as by FORTRAN program using algorithms in Numerical Recipes [42]. The noise term was calculated as an independent Gaussian random number. We confirmed the accuracy by reducing the time grid for integration.

## Supporting Information

Text S1 Supporting text for this paper.
(0.04 MB PDF)

Figure S1 Adaptive Response of the Network in Cells with pALL8. (A) The structure of pALL8 is the same as pALL7 shown in Figure 1 except for the exchanged positions of *mdhfr* and *gls-h*. Ptrc and PtetA represent the *trc* promoter and *tetA* promoter, respectively. (B) The adaptive shifts in gene expression of pALL8 that are opposite to those of pALL7. Cells carrying pALL8 were subjected to a series of overnight cultures in the same manner as in Figure 4 with a slightly modified media as follows. As trimethoprim lactate at the concentration used in the original Medium T completely suppressed the growth of cells, it was diluted to 3 mg/l. To further support the cells with trimethoprim lactate, we increased the anhydrotetracycline concentration from 0.5 µg/l to 0.8 µg/l to achieve higher levels of expression of mDHFR (Medium T′). The same concentration of anhydrotetracycline was used in the other two media (Medium M′ and N′) for accurate comparison. Cells grown in Medium N′ were subjected to a series of overnight cultures with an inoculum size of 7×107 cells/l every day in Medium N′ for 4 days (blue), in Medium T′ for 4 days (green), and in Medium M′ for 7 days (red) in the same way as described in Figure 4.
(0.01 MB PDF)

Figure S2 Flow Cytometric Analysis of Attractor Selection by Changing Environments. (A) The original data from the flow cytometry results shown in Figure 4A. (B) The original data for Figure 4B. For each culture, 10,000 events were collected. The weak positive correlation observed in Medium T was due to leakage of the red fluorescence to the green fluorescence gate and was not related to GFP expression. Note that when cells located around Attractor 1 in Medium M were transferred to Medium T (upper right panel in B), a small fraction of cells stayed in this attractor and exhibited the same scattering of fluorescence intensity, indicating gene expression noise in this non-adaptive state.
(0.05 MB PDF)

Figure S3 Basic Characteristic of the Model in Equation (1). Phase space spanned by *m*1 and *m*2 with nullclines, d*m*1/d*t* = 0 or d*m*2/d*t* = 0 in Eq. (1), calculated for (*N*1,*N*2) = (0,10) in Eq. (2), by adopting S(*A*) = 6*A*/(2+*A*) and *D*(*A*) = *A* (solid lines) with the parameter values *P* = *C* = 0.01, *N*_thr_1_ = *N*_thr_2_ = 2, *n*_1_ = *n*_2_ = 5. The separatrix (dashed line) is given by *m*1 = *m*2. The fixed-point attractors are given by the intersections of the two solid lines (filled circles).
(0.03 MB PDF)

Figure S4 Bifurcation Diagram of the Model with Equations (1) and (2). Bifurcation diagram for d*m*1/d*t* = 0 and d*m*2/dt = 0 in Eq. (1) for the same condition as in Figure S3 (solid line). Since *m*2 = 1/*m*1 for *A*<1 and *m*2 = *m*1 for *A*>1 are adopted as steady-state(s), only *m*1 (horizontal axis) is indicated as a function of change of the parameter *A* (vertical axis). The bifurcation point (*m*1,*m*2) = (1, 1), as well as the adaptive attractor with large and the non-adaptive attractor with small are represented by the three filled circles. The dependency of *A* on (steady-state) is computed from the condition d*A*/d*t* = 0 in Eq. (2) (broken line) where *m*2 = 1/*m*1 is adopted as *A*<1.
(0.02 MB PDF)

Figure S5 Dependence of the Fraction of Selection of Adaptive Attractor on the Noise Strength for the Model (1)–(2). The frequency the cell is in the adaptive attractor is plotted against the standard deviation of the noise per single time step. For each noise strength, 10,000 runs of simulation were performed by changing the environment at the time step 2,000, while the frequency of adaptive attractor selection is computed from the number of runs that ended with *m*1>*m*2 at the time step 20,000. The vertical axis represents the ratio of such runs to the total number of runs, 10,000.
(0.25 MB PDF)
