# Supplementary material for: Adaptive Response of a Gene Network to Environmental Changes by Fitness-Induced Attractor Selection
Source: PLoS One. 2006 Dec 20;1(1):e49. doi: 10.1371/journal.pone.0000049 (PMC1762378; doi:10.1371/journal.pone.0000049)
Supplement: Text S1 — Supporting text for this paper. (0.04 MB PDF) [file pone.0000049.s002.pdf]

Supporting Information:

1. Model: incorporating cellular activity *A*

Here, we give detailed simulation results for the model expressed in equation (1). First, we set $D(A)=A$, assuming that cell growth rate, which accounts for dilution and hence, decrease rate of the gene products, D, is roughly propotional to activity, and $S(A)=\alpha A/(\beta + A)$, since the increase of synthesis with activity usually exhibit some threshold behavior and saturates. If S/D exceeds 0.5 with the decrease of A, bifurcation from the monostable behavior with a single Attractor W to the bistable behavior occurs (see Figure S4), as observed in the experiment. For parameter values, α =6 and β =2, i.e., $S(A)=6A/(2+A)$ and $D(A)=A$, the presented result is valid as long as S/D >0.5 for small A and <0.5 for large A. With this choice, Eq. (1) generate a single attractor $m1=m2$ (corresponding to Attractor W in the experiment) for A>1, whereas they give rise to the two attractors satisfying $m1=\frac{1}{m2}$ with separatrix of $m1=m2$ (see Figure S3) for A<1. The total abundance of mRNAs, $m1+m2$, is positively correlated with the ratio of the synthesis rate to the dilution rate, $S(A)/D(A)$. As the mutually inhibitory operons can keep a high concentration of only one of the two mRNAs, the network will exhibit strong expression of either one of the two operons at $S(A)/D(A)$ >0.5. As long as $S(A)/D(A)$ or the total mRNA level increases with A, other mathematical forms of $D(A)$ and $S(A)$ will yield essentially the same result. Indeed, mRNA quantification (see main text) confirmed an increase by one-order of magnitude in the total mRNA level through the environmental change from rich (Medium N) to the poor, selective medium (Medium M or T).

Cell activity is correlated with cell growth rate and the nutrition condition. In the experiment, to maintain activity, either nutrient1 (glutamine) and $m1$ must coexist or

nutrient 2 (tetrahydrofolate) and $m2$. Such dynamics of A, indeed, is given essentially by the classic model for cell growth rate [1],

$$\frac{d}{dt}A = \frac{P}{((\frac{N\_thr_1}{m1+N1})^{n_1}+1)\times((\frac{N\_thr_2}{m2+N2})^{n_2}+1)} - C\times A \quad (2)$$

where P and C are the rate coefficients of the production and consumption of activity, respectively. $N1$ and $N2$ represent the external supplementation of the two nutrients, which were also supplied by expression of the enzymes encoded by Operons 1 and 2, respectively, while N_thr_1 and N_thr_2 represent the thresholds of the two nutrients to the production of A, and n_1 and n_2 are the sensitivities (Hill coefficients) of the two nutrients.

2. Simulation

We have carried out several simulations of the above model, using for simplicity the same values for the two parameters with subscripts of 1 and 2 (but again this choice is not necessary). When $N1$ and $N2$ are sufficiently high, the nutritious load given by the denominator of the first term of (2) approaches its minimum and A approaches its upper limit, P/C (*e.g.*, one under the conditions shown in Figure 6). With this high activity value A, the network is around the bifurcation point or within the region of a single attractor (Figure S3).

Depletion of one of the nutrients, *e.g.*, $N1=0$, results in the nutritious load becoming large to reduce activity A, bringing the network into the region of two attractors (Figure S4). Under these conditions, the nutritious load is dependent on the expression level of mRNA ($m1$) that compensates for the nutrient depletion so that the stationary value of activity satisfying $dA/dt=0$ is an increasing function with respect to $m1$ (Figure S4).

Thus, the attractor with strong expression of $m1$, which compensates for the depletion of $N1$, can maintain a large value of A, and is defined as adaptive, while the other attractor having a very low value is non-adaptive.

We next asked whether the network chooses the adaptive over the non-adaptive attractor on transfer from the environment containing sufficient levels of $N1$ and $N2$ ($A \sim 1$) to the environment depleted of either nutrient ($A<1$). Although the two attractors differ in activity, they possess symmetrical basins with separatrix of $m1 = m2$ (Figure S3). Therefore, depending on the expression immediately after the transfer, the network could move into either of the two attractors by chance. However, the numerical simulation with equations (1), and (2) confirmed that the network chooses the adaptive attractors on encountering conditions of nutrient depletion (Figure 6). We also found that particle simulation using Gillespie's method supports the present result (Furusawa and Kaneko, unpublished).

The simulations confirm that adaptive attractor selection is explained by our general argument in the main text: For the attractor with lower activity A, the dynamics is mainly governed by noise, while when the system reaches the gene expression state capable of compensating for nutrient depletion, the activity is increased so that the deterministic terms work again to bring the network eventually to the adaptive attractor. Therefore, selection of the adaptive over the non-adaptive attractor is a consequence of the cooperation of noise and A. Indeed, when too little noise is applied to the simulation, the two attractors appear with the same frequency, regardless of which nutrient is depleted (Figure S5). Note also that the symmetry between $m1$ and $m2$ in Eq. (1) is not necessary for selection of the adaptive attractor selection. The selection works even if the terms for $m1$ and $m2$ in Eq. (1) include different parameter values, as long as they

exhibit bistable behavior. In Figure S5, the frequency of selection of the adaptive attractor is plotted against the size of noise. If the noise intensity is too small the selection of each attractor occurs with equal probability due to the symmetry of the deterministic saddle between the two attractors. The levels of $m1$ and $m2$ are not sufficient to cross the separatrix. For very large noise intensity, the selection occurs again with equal probability, since the dynamics are governed by the noise term alone that is so large that the separatrix is crossed. The mechanism for adaptive attractor selection works for intermediate noise intensity, where the selection probability of the adaptive attractor is almost unity.

1. Nielsen J, Villadsen J (1994) Bioreaction Engineering Principles. New York: Plenum Press. 480 p.
