# Supplementary figures and images for: Adaptive Response of a Gene Network to Environmental Changes by Fitness-Induced Attractor Selection
Source: PLoS One. 2006 Dec 20;1(1):e49. doi: 10.1371/journal.pone.0000049 (PMC1762378; doi:10.1371/journal.pone.0000049)

A

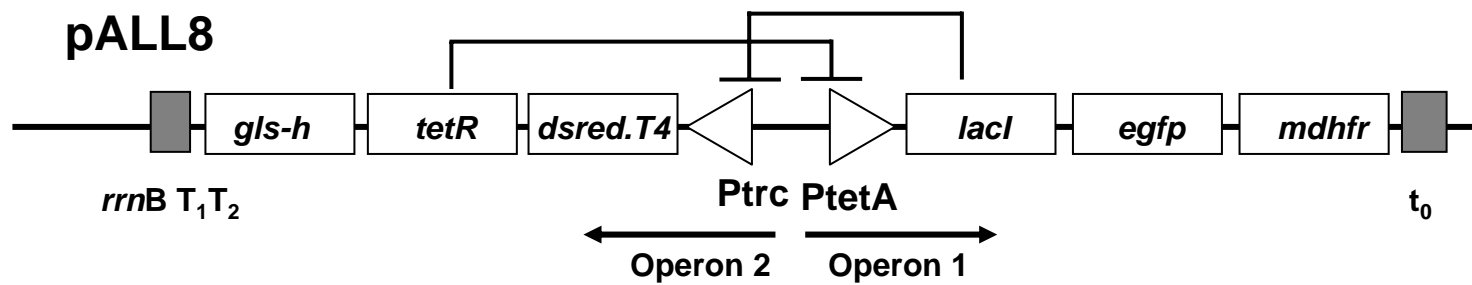

B

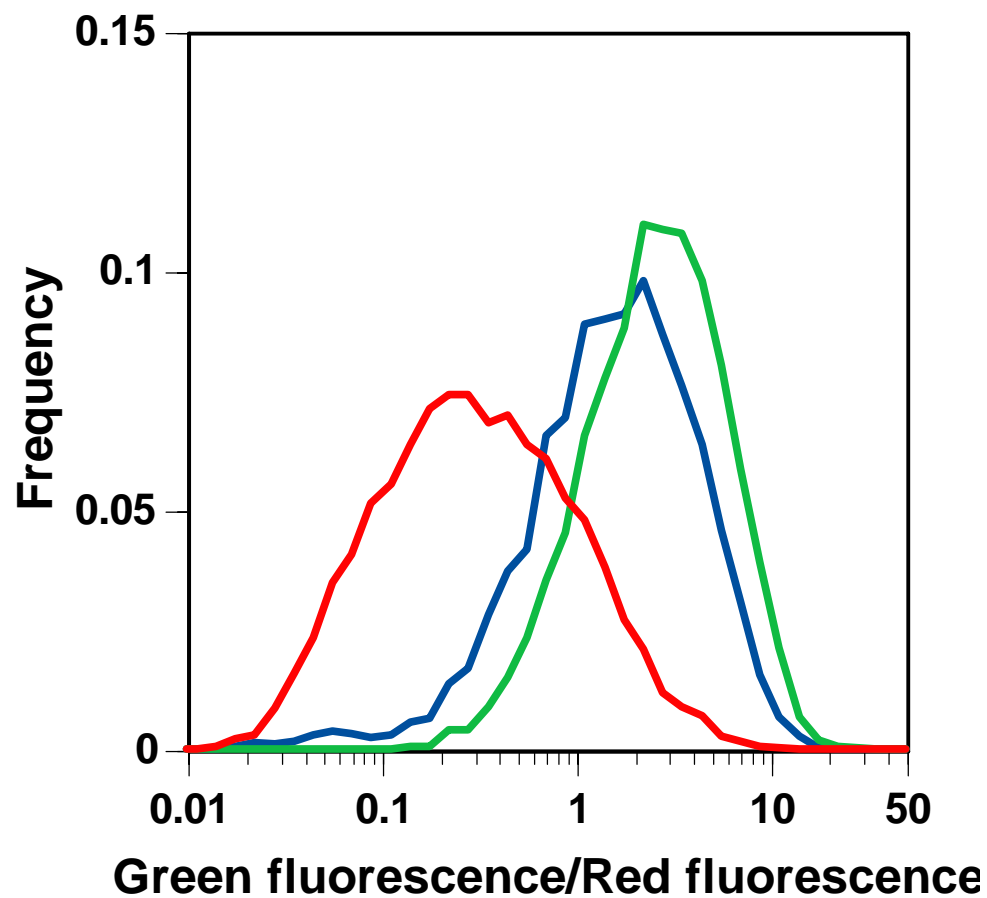

Supplement: Figure S1 — Adaptive Response of the Network in Cells with pALL8. (A) The structure of pALL8 is the same as pALL7 shown in Figure 1 except for the exchanged positions of mdhfr and gls-h. Ptrc and PtetA represent the trc promoter and tetA promoter, respectively. (B) The adaptive shifts in gene expression of pALL8 that are opposite to those of pALL7. Cells carrying pALL8 were subjected to a series of overnight cultures in the same manner as in Figure 4 with a slightly modified media as follows. As trimethoprim lactate at the concentration used in the original Medium T completely suppressed the growth of cells, it was diluted to 3 mg/l. To further support the cells with trimethoprim lactate, we increased the anhydrotetracycline concentration from 0.5 µg/l to 0.8 µg/l to achieve higher levels of expression of mDHFR (Medium T′). The same concentration of anhydrotetracycline was used in the other two media (Medium M′ and N′) for accurate comparison. Cells grown in Medium N′ were subjected to a series of overnight cultures with an inoculum size of 7×10^7^ cells/l every day in Medium N′ for 4 days (blue), in Medium T′ for 4 days (green), and in Medium M′ for 7 days (red) in the same way as described in Figure 4. (0.01 MB PDF) [file pone.0000049.s003.pdf]

**A**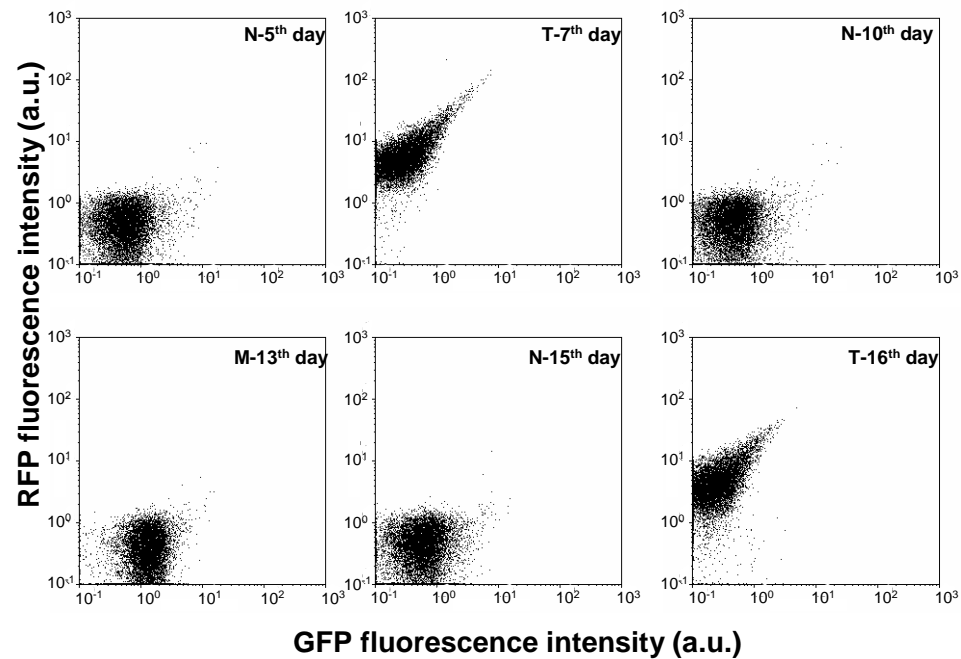**B**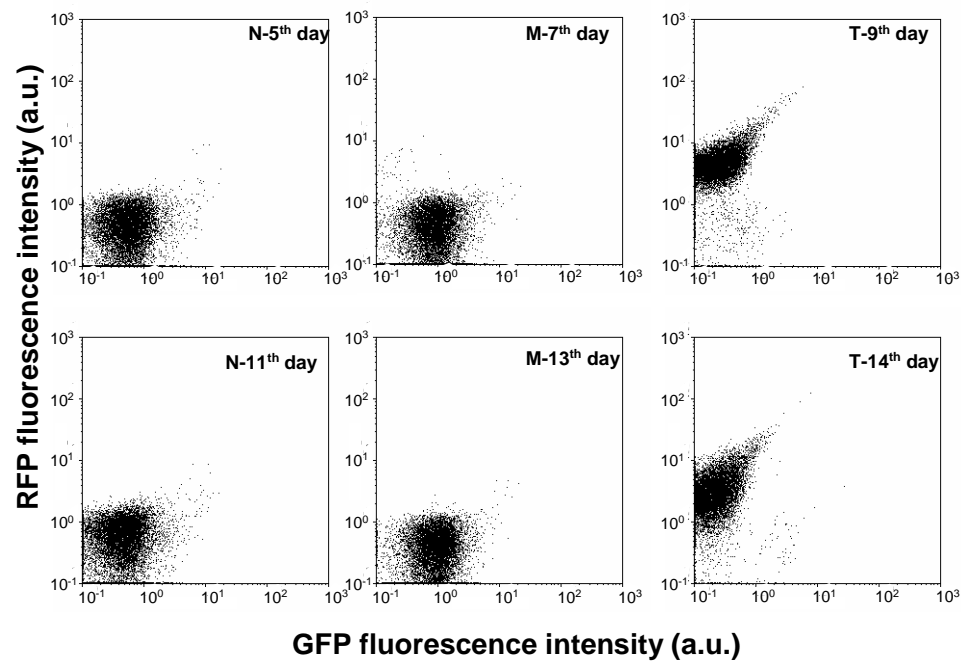

Supplement: Figure S2 — Flow Cytometric Analysis of Attractor Selection by Changing Environments. (A) The original data from the flow cytometry results shown in Figure 4A. (B) The original data for Figure 4B. For each culture, 10,000 events were collected. The weak positive correlation observed in Medium T was due to leakage of the red fluorescence to the green fluorescence gate and was not related to GFP expression. Note that when cells located around Attractor 1 in Medium M were transferred to Medium T (upper right panel in B), a small fraction of cells stayed in this attractor and exhibited the same scattering of fluorescence intensity, indicating gene expression noise in this non-adaptive state. (0.05 MB PDF) [file pone.0000049.s004.pdf]

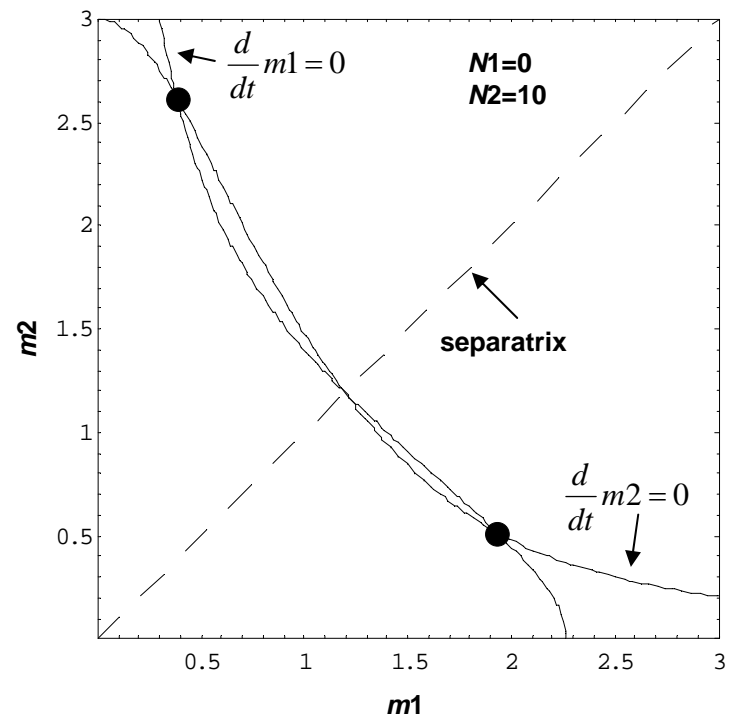

Supplement: Figure S3 — Basic Characteristic of the Model in Equation (1). Phase space spanned by m1 and m2 with nullclines, dm1/dt = 0 or dm2/dt = 0 in Eq. (1), calculated for (N1,N2) = (0,10) in Eq. (2), by adopting S(A) = 6A/(2+A) and D(A) = A (solid lines) with the parameter values P = C = 0.01, N_thr1 = N_thr2 = 2, n 1 = n 2 = 5. The separatrix (dashed line) is given by m1 = m2. The fixed-point attractors are given by the intersections of the two solid lines (filled circles). (0.03 MB PDF) [file pone.0000049.s005.pdf]

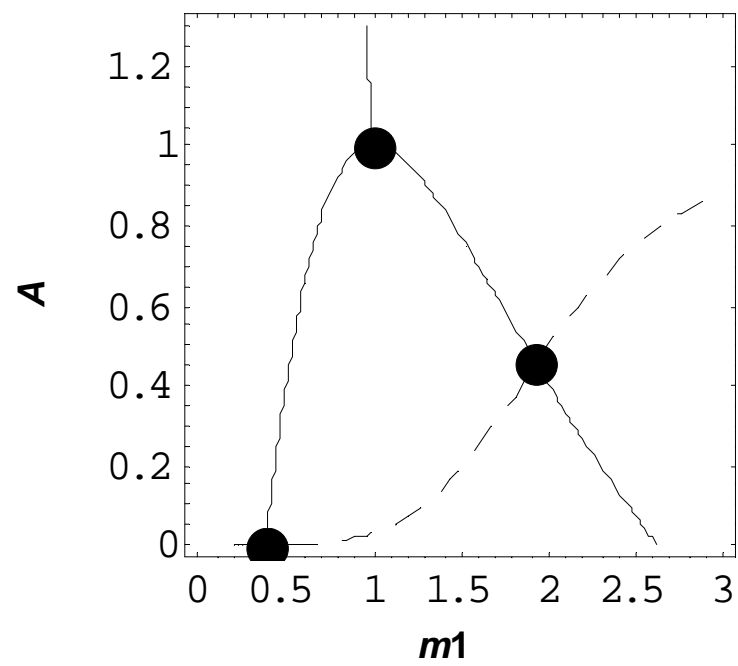

Supplement: Figure S4 — Bifurcation Diagram of the Model with Equations (1) and (2). Bifurcation diagram for dm1/dt = 0 and dm2/dt = 0 in Eq. (1) for the same condition as in Figure S3 (solid line). Since m2 = 1/m1 for A<1 and m2 = m1 for A>1 are adopted as steady-state(s), only m1 (horizontal axis) is indicated as a function of change of the parameter A (vertical axis). The bifurcation point (m1,m2) = (1, 1), as well as the adaptive attractor with large and the non-adaptive attractor with small are represented by the three filled circles. The dependency of A on (steady-state) is computed from the condition dA/dt = 0 in Eq. (2) (broken line) where m2 = 1/m1 is adopted as A<1. (0.02 MB PDF) [file pone.0000049.s006.pdf]

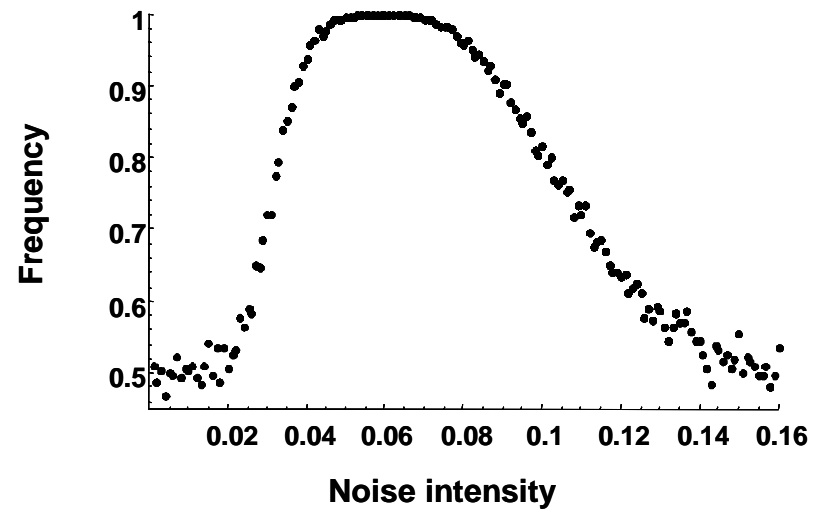

Supplement: Figure S5 — Dependence of the Fraction of Selection of Adaptive Attractor on the Noise Strength for the Model (1)–(2). The frequency the cell is in the adaptive attractor is plotted against the standard deviation of the noise per single time step. For each noise strength, 10,000 runs of simulation were performed by changing the environment at the time step 2,000, while the frequency of adaptive attractor selection is computed from the number of runs that ended with m1>m2 at the time step 20,000. The vertical axis represents the ratio of such runs to the total number of runs, 10,000. (0.25 MB PDF) [file pone.0000049.s001.pdf]
